# Supplementary material for: Are carbon emissions trading and green financial instruments synergistic? -Comprehensive quantitative research based on content analysis
Source: PLoS One. 2024 Mar 7;19(3):e0298601. doi: 10.1371/journal.pone.0298601 (PMC10919601; doi:10.1371/journal.pone.0298601)
Supplement: S1 Data — (ZIP) [file pone.0298601.s001.zip › Data/地级市Z_F工作报告.zip!/╣π╢1⁄2/╔ε█┌╩╨/└┤╫╘╣1⁄2╓┌║┼í╛┬φ┐╦╩2╛▌═°í┐_2015.docx]

各位代表：

　　现在，我代表深圳市人民政府向大会作政府工作报告，请予审议，并请各位政协委员和其他列席人员提出意见。

　　一、过去五年工作回顾

　　市五届人大一次会议以来的五年，是深圳经济特区在新三十年起点上，实现发展理念重大转变、发展内涵显著跃升、发展动力持续增强的五年，是成功应对各种风险挑战，化危为机、逆势而进，取得改革发展巨大成就的五年。在党中央国务院、省委省政府和市委的正确领导下，全市上下深入贯彻落实党的十八大、十八届三中、四中全会和习近平总书记系列重要讲话精神，围绕“三个定位、两个率先”目标，坚持“三化一平台”改革主攻方向，牢固树立“深圳质量”新理念，着力推动有质量的稳定增长、可持续的全面发展，完成了经济社会发展主要目标任务，为深圳经济特区迈向“四个全面”新征程打下了坚实基础。

　　2014年本市生产总值超过1.6万亿元，提前实现“十二五”规划1.5万亿元的目标，总量五年翻了近一番；固定资产投资2717亿元，五年累计突破1万亿元，超过此前十年总和；社会消费品零售总额4844亿元，是五年前的1.9倍；进出口总额4878亿美元，连续三年居内地城市首位，其中出口2844亿美元，实现二十二连冠；来源于我市的公共财政收入5560亿元，五年翻了一番，其中地方一般公共预算收入2082亿元，是五年前的2.4倍。

　　五年来，我们主动创新发展理念，把质量作为第一追求，推动经济社会发展率先迈入质量时代。按照中央关于把发展的立足点转到提高质量和效益上来的要求，我们在“深圳速度”的基础上，提出了“深圳质量”“深圳标准”的新理念和新要求，着力推进标准、质量、品牌、信誉“四位一体”建设，加快转变经济发展方式，全面提升发展质量。五年来，质量理念转化为质量意识，内化为质量自觉，成为社会普遍认同的价值观，贯穿到经济社会发展各领域和全过程。深圳获批成为首个全国质量强市示范城市。质量高、结构优、创新强、速度稳成为深圳经济特区发展的新常态。发展的“价值含量”显著提升。人均GDP五年增加1万美元，达到2.4万美元，每平方公里产出GDP、财政收入分别达8亿元和2.8亿元，均居全国大城市首位。工业增加值率五年提高4.9个百分点。地方一般公共预算收入年均增速是GDP增速的1.8倍。居民人均可支配收入、最低工资标准、最低生活保障标准均居全国领先水平。发展的“技术含量”显著提升。全社会研发投入达到643亿元，是五年前的2.3倍，占GDP的4.02%。战略性新兴产业年均增长20%以上，增加值占GDP比重超过35%，对经济增长的贡献率接近50%。国家级高新技术企业4742家，五年新增3698家。发展的“绿色含量”显著提升。万元GDP能耗、水耗五年累计分别下降19.5%和44.7%，化学需氧量、氨氮、二氧化硫减排量提前两年超额完成“十二五”目标，PM2.5平均浓度降至33.6微克/立方米，空气质量居副省级以上城市最优水平，以更少的资源能源消耗、更低的环境成本支撑了更高质量、更可持续的发展。在2014年珠三角“九年大跨越”考核中深圳获全省第一名。

　　五年来，我们变压力为动力、化被动为主动，不失时机加快结构调整，成功实现新一轮转型升级。面对国际金融危机的冲击，我们迎难而上，全力推进新一轮转型升级。即使在增速短期下滑的压力下，始终保持目标不变、力度不减、步伐不停，坚持区域转型和产业升级并举，大力推动增量优质、存量优化，五年累计淘汰转型低端落后企业超过1.6万家，以转型升级的主动实现腾笼换鸟、凤凰涅槃。梯次型现代产业体系基本形成。实施生物、互联网、新能源、新材料、文化创意、新一代信息技术、节能环保等七大战略性新兴产业规划政策，深圳成为国内战略性新兴产业规模最大、集聚性最强的城市，产业总规模近2万亿元。前瞻布局航空航天、生命健康、机器人、可穿戴设备和智能装备等未来产业，不断形成新的优质产业增量。服务业占GDP比重达57.3%，五年提高4.1个百分点，其中现代服务业占比达67.6%。服装、家具、钟表等优势传统产业加速向微笑曲线两端攀升，黄金珠宝产业集聚基地等成为全国知名品牌创建示范区，工业设计从业人员约占全国1/4，IF国际设计大奖和红点奖获奖总数连续四年居全国首位。产业集聚辐射能力显著增强。总部经济影响力持续扩大，华为等4家本土企业进入世界500强。金融中心地位不断巩固，本外币存贷款余额跃居全国第三，金融业总资产突破7万亿元，五年翻了一番。在境内外上市的深圳企业达306家，五年新增113家。港口集装箱年吞吐量超过2400万标箱，进入全球前三。机场旅客年吞吐量超过3600万人次。华星光电8.5代液晶面板、长安标致雪铁龙汽车等重大项目建成投产。电子商务年交易额突破1.5万亿元，占全国比重超过1/9。中国驰名商标达143个，五年增长2倍。新的区域增长极加快形成。深圳湾超级总部基地、国际生物谷等15个重点区域开发建设加快，留仙洞等23个战略性新兴产业基地和集聚区成为新兴产业发展的重要载体，盐田综合保税区获批，光明、坪山、龙华、大鹏等四个功能新区成为各具特色的区域增长极。

　　五年来，我们把创新摆在与改革开放同等重要位置，积极构建综合创新生态体系，创新主引擎作用更加突出。在激烈的国际竞争中，惟创新者进，惟创新者强，惟创新者胜。深圳全面落实创新驱动发展战略，深入实施国家创新型城市总体规划，出台创新驱动发展“1＋10”文件，加快向引领式创新和全面创新迈进，成为首个以城市为基本单元的国家自主创新示范区。重大创新成果不断涌现。2014年PCT国际专利申请量达1.16万件，是五年前的3倍，占全国的48.5%。每万人有效发明专利拥有量65.7件，是全国平均水平的13.4倍。第四代移动通信、基因测序、超材料、新能源汽车等领域技术水平居全球前列。深圳的科研成果和科技人员分别入选《科学》杂志年度全球十大科学突破、《自然》杂志年度全球科学界十大人物。五年累计获国家技术发明一等奖、科技进步一等奖等国家科学技术奖励50项。创新能力建设实现重大跨越。国家超级计算深圳中心、国家基因库和大亚湾中微子实验室建成，国家、省和市级重点实验室、工程实验室、工程研究中心和企业技术中心等创新载体累计达1107家，是前三十年总和的3.6倍。华大基因、光启等新型研发机构呈现引领式创新、爆发式增长态势。五年累计引进“孔雀计划”“珠江人才计划”创新团队59个。三星通信设备研究院等一批跨国公司研发中心落户深圳。综合创新生态体系更加完善。推进国家科技金融试点城市建设，出台支持互联网金融、股权投资发展等政策措施，VC/PE机构达1.5万家、注册资本超过1.1万亿元。组建45个高水平产学研资联盟和7个专利联盟。国家技术转移南方中心落户深圳，科技服务业规模超过900亿元。设立深圳国际创客周，实施青少年创新支持计划，柴火空间、创客市集等一批众创空间快速兴起，草根创新、万众创新蓬勃发展。高交会、电子信息博览会、国际人才交流大会、国际工业设计大展、IT领袖峰会和BT领袖峰会等成为促进创新创业的重要平台。

　　五年来，我们始终坚持改革不停顿、开放不止步，紧紧围绕“三化一平台”，着力以改革破难题，以开放促发展。改革开放永无止境，停顿倒退没有出路。我们始终牢记特区使命，深入落实十八届三中全会精神和国务院批复的综合配套改革总体方案，主动承接国家、省242项改革任务，加大改革攻坚力度，进一步激发了发展活力。重点领域和关键环节改革取得新突破。在全国率先实施商事制度改革，推行“四证合一”，商事主体从68.3万户增加到185.8万户，总量居全国城市首位。改革投融资体制和社会投资管理体制，社会投资项目核准事项压减90%，社会投资占比从64.6%提高到80.2%。开展新一轮土地管理制度改革试点，存量建设用地占土地供应总量的比重提高到70%。实施第四轮财政体制改革，启动政府全口径预决算管理。积极推进“营改增”，试点纳税人总体税负下降40.3%。深化国资国企改革，市属国企混合所有制比例超过75%，国有净资产增长140%。公立医院管办分离、医药分开改革取得重大进展，率先全面实施药品“零加成”。积分入户政策全面实行。公务员分类管理和聘任制、社会组织管理体制、巨灾保险等改革走在全国前列，教育、养老服务业等改革取得积极进展。前海战略平台效应加速释放。国务院批复的前海总体发展规划和22条先行先试政策加快落地，基础设施建设、重点单元开发全面推进，注册企业超过3万家、注册资本超过1.8万亿元，要素交易平台达19家。首家民营互联网银行前海微众银行开业，跨境人民币贷款率先破冰，国际仲裁院、廉政监督局、前海法院挂牌成立。出台前海促进深港合作工作方案，深港青年梦工场建成运营，港企对前海GDP贡献率超过1/5。开放型经济水平持续提升。广东自贸区前海蛇口片区挂牌。粤港澳大湾区纳入国家“一带一路”愿景与行动，制定实施发展湾区经济、建设海上丝绸之路桥头堡的若干意见。着力优化外贸结构，全面完成来料加工贸易企业不停产转型，一般贸易、技术贸易额分别是五年前的2倍和5倍，服务贸易规模突破1000亿美元。合同利用外资、对外直接投资额分别增长2.1倍和12.6倍。成立深圳国际交流合作基金会。国际友城和友好交流城市达65个，五年增加一倍。深莞惠经济圈建设提速，全面对口帮扶河源、汕尾和扶贫开发“双到”工作深入推进，深汕特别合作区建设加快，援疆、援藏、对口帮扶贵州取得新成效。

　　五年来，我们牢固树立环境就是生产力、竞争力的理念，率先走出绿色低碳发展新路径，环境价值进一步凸显。积极落实建设美丽中国重大部署，出台推进生态文明、建设美丽深圳的决定，率先实施低碳发展中长期规划和生态文明建设考核，率先建立环境形势分析会制度，推进大气环境、水环境和绿化美化三大工程，城市绿色颜值显著提升，市民绿色福利不断增多。节能减排成效明显。在全国率先全面实施绿色建筑标准，绿色建筑总面积超过2100万平方米，规模居全国城市首位，综合节能总量累计超过410万吨标准煤。全市机关事业单位建筑面积一半以上完成节能改造，年节电超过1亿度。妈湾电厂六台机组全部完成脱硫脱硝改造。累计淘汰黄标车、老旧车17.3万辆。全面推广使用国Ⅴ汽柴油。试点推广垃圾减量分类。盐田区餐厨垃圾处理等一批项目获“中国人居环境范例奖”。化学需氧量、氨氮、二氧化硫和氮氧化物排放五年累计分别下降45.8%、37.0%、43.5%和23.8%。低碳发展持续推进。开展碳排放权交易试点，管控单位碳排放强度明显下降。累计推广应用新能源汽车超过1万辆，居全球城市前列。国际低碳城成为中欧可持续城镇化合作旗舰项目，中美低碳建筑与社区创新实验中心等一批项目成功入驻。生态环境更加优美。累计新建污水管网1280公里、污水处理厂15座，污水处理能力扩大一倍。龙岗河、坪山河等跨市河流治理通过省人大评估考核，茅洲河等综合整治全面启动。深圳获评为“国家节水型城市”。建成475公里、8361公顷生态景观林带，绿道总长2400公里。新建、改造公园236个，公园总数达889个，深圳湾公园、大鹏半岛国家地质公园、盐田海滨栈道等成为靓丽的湾区风景线。

　　五年来，我们抓住大运会举办、特区范围扩大等重大机遇，推动有质量的深度城市化，城市现代化国际化水平实现新跃升。认真落实国家新型城镇化战略，出台提升城市发展质量决定和国际化城市建设行动纲要，连续实施两轮特区一体化三年计划，城市综合服务功能更加完善。大运会显著提升城市国际形象。成功举办第26届世界大学生夏季运动会，赛会规模和项目数量创历届之最，创造了“不一样的精彩”，极大提升了深圳的国际影响力、城市凝聚力和市民自豪感。特区一体化优化城市空间开发格局。制定实施新一轮城市总体规划、土地利用总体规划，组团式空间布局进一步完善。原特区外投资五年累计超过8000亿元，占全市投资70%以上。五年整备土地106平方公里，实施城市更新项目419个。建成深圳湾体育中心、机场新航站楼等一批新地标，42个项目获鲁班奖、国家优质工程奖等国家级大奖。现代化基础设施增强城市功能。深圳北站、广深客运专线、厦深铁路等重大基础设施投入使用，地铁运营和在建里程达350公里，公交日均客流量突破千万人次。“七横十三纵”干线路网架构基本形成，沿江高速、博深高速、新彩通道等建成通车，外环高速、坪盐通道、坂银通道、深圳东部过境高速等一批重大交通基础设施开工。梧桐山隧道、盐田坳隧道、梅观高速深圳段等收费取消，打通断头路125条。智慧城市建设深入推进，互联网、家庭宽带、无线宽带覆盖率分别达85%、85.2%和89%，重要公益性公共场所初步实现WiFi免费接入。资源能源保障能力进一步提高，西气东输二线正式供气，清林径引水、公明供水调蓄工程、抽水蓄能电站等进展顺利，燃气管网覆盖率提高27.6个百分点。精细化管理提高城市安全水平。全面开展公共安全风险评估，率先发布实施城市公共安全白皮书。成功创建国家防震减灾示范城市。食品药品监管持续加强，抽检总量五年提高一倍。推进平安深圳建设，构建治安立体防控体系，110刑事治安总警情数下降50.4%。全面实行安全生产“一岗双责”，加快完善隐患排查治理体系，开展道路交通、泥头车、地面塌陷、易涝点等专项治理，亿元GDP生产安全事故死亡率下降60.9%。

　　五年来，我们把增进民生福祉作为根本目的，多层次、系统化推进民生事业跨越发展，发展成果更多更好惠及市民。出台关于加强社会建设的决定，大力引进教育、医疗等优质资源，实施12项跨年度重大民生工程，扎实办好年度民生实事，五年全市财政九类重点民生领域投入近5000亿元，年均增长22.6%。基本民生保障水平持续提升。最低生活保障标准从415元提高至800元，最低工资标准从1000元提高至2030元。社会保险参保总人次五年增长58%。租售补相结合的住房保障体系基本形成，保障房开工21.7万套、竣工9.04万套、供应9.1万套，惠及人才和低收入群体超过60万人。新增养老床位4098张，建成老年人日间照料中心25家。教育事业实现新发展。出台中长期教育改革和发展规划纲要，教育投入年均增长22.2%。新建普惠性幼儿园612所，新增幼儿园学位13.8万个、中小学学位12.6万个。高等教育开放式跨越发展，南方科技大学、香港中文大学（深圳）正式招生，深圳北理莫斯科大学、清华-伯克利深圳学院、深圳吉大昆士兰大学启动筹建，深圳墨尔本生命健康工程学院、湖南大学罗切斯特理工学院、华大基因学院、国际太空科技学院等一批特色学院建设加快。医疗卫生事业迈出新步伐。鼓励社会资本举办三级医院，实施医疗卫生“三名工程”，引进24个国内外一流医疗团队，新增医院20家，千人病床数增长37.2%。三甲医院由3家增至10家，国家级医学重点学科由4个增至12个。香港大学深圳医院、内地首家香港独资眼科医院等正式运营，市人民医院外科楼、儿童医院住院大楼等重大卫生项目建成启用。家庭医生试点稳步推进。文化体育等社会事业取得新进步。深圳被联合国教科文组织授予“全球全民阅读典范城市”称号，连续四次蝉联“全国文明城市”，连续五次获“全国双拥模范城”称号。当代艺术馆与规划展览馆等一批重大文体设施启动建设，《走向复兴》等12部作品获国家“五个一工程”奖，《人文颂》在联合国总部成功演出。ATP、WTA国际网球公开赛、深圳国际马拉松赛、中国杯帆船赛等高水平国际赛事成功举办。文化创意产业增加值1560亿元，年均增长22%。旅游年度总收入超千亿元，年接待游客突破1亿人次。实施“织网工程”和“风景林工程”，率先建立公共信息资源库，建成社区服务中心538个，社会组织超过8600家，专业社工达7600人，注册志愿者105万。中国慈展会落户深圳。此外，信访、统计、计生、司法、民族、宗教、对台、侨务、口岸、气象、民防、档案等工作都取得了新成绩。

　　五年来，我们突出为民务实清廉，深入推进职能和作风“两个转变”，政府自身建设进一步加强。开展党的群众路线教育实践活动，加快建设法治政府、廉洁政府和服务型政府，行政效能持续提升。依法行政水平不断提高。自觉接受市人大、市政协监督，认真执行法律法规和市人大各项决议决定，建立向人大报告和向政协通报工作的常态化机制，五年办理市人大代表建议3303件、市政协提案2678件。提请市人大审议法规议案42件，新制定政府规章53项，修订、废止政府规章67项。法治政府建设指标体系、政府法律顾问制度获“中国法治政府奖”。简政放权力度加大。编制政府部门权责清单，出台转变政府职能事项和政府购买服务目录。深化行政审批制度改革，精简行政审批事项205项，清理幅度达47%。优化部门审批流程，市政府投资项目、重大社会投资项目审批实现跨部门协同办理。市区两级网上办事大厅全部开通，行政审批事项99%实现网上申报。作风建设持续加强。严格落实中央八项规定，坚决反对“四风”，“三公”经费大幅压减，公车改革顺利实施。加大服务企业力度，实施领导干部挂点联系企业和重大项目分级协调制度。加强行政监察、审计监督和政府绩效考核管理，廉政建设和反腐败工作取得新成效。

　　各位代表，过去五年，我们在历届市领导班子和一代又一代特区建设者打下的坚实基础上，继承中创新、发展中突破，取得了来之不易的成绩。这主要得益于我们坚定不移地贯彻落实党中央国务院、省委省政府和市委的决策部署，不断探索中国特色、时代特征、深圳特点的发展之路，以特区的担当践行国家战略，以特区的作为服务发展大局，以特区的实践彰显中国特色社会主义制度的优越性；得益于我们坚定不移地走有质量、可持续发展之路，牢固树立质量第一的理念，前瞻规划、科学布局，抢先培育发展战略性新兴产业和未来产业，扎实推进创新驱动、转型升级、绿色低碳，以质量提升实现稳定增长、增强发展后劲，推动特区发展行稳致远；得益于我们坚定不移地以改革破难题促发展，坚持社会主义市场经济改革方向，既发挥好市场内在牵引、利益驱动作用，又更好地发挥政府规划引领、定向调控作用，形成有效市场、有为政府的有机统一，使资源配置更加高效、发展更有活力；得益于我们坚定不移地把保障和改善民生作为工作的出发点和落脚点，以经济发展促进民生改善，将发展成果更多用于民生之需，不断提升民生幸福水平；得益于全市人民齐心协力，团结拼搏，以辛勤劳动创造美好生活，以创新创业铸就特区辉煌。在这里，我代表深圳市人民政府，向全市人民，向给予政府工作大力支持的人大代表和政协委员，向各民主党派、工商联、各人民团体和社会各界人士，向中央和省各部门、中央和各省区市驻深单位，向驻深解放军、武警官兵，向关心和支持深圳发展的港澳同胞、台湾同胞、海外侨胞和国际友人，表示崇高的敬意和衷心的感谢！

　　与此同时，我们也清醒地认识到发展中还存在一些突出问题、面临不少挑战。一是率先全面建成小康社会还有一些薄弱环节，经济社会发展不够协调，教育、医疗资源总量不足、分布不均，特区一体化和基本公共服务均等化任务十分繁重；二是人才总量与结构还不适应新常态下动力转换、结构优化的需要，创新型人才、高技能人才的引进培养力度需进一步加大；三是改革的系统性、整体性、协同性有待增强，破解深层次矛盾难度加大，改革攻坚锐气有所弱化；四是城市管理面临较大压力，违法建筑问题突出，部分区域环境卫生管理不到位，垃圾减量分类进展较慢，公交便利化程度还不高，食品药品安全保障能力亟需加强；五是生态文明建设任务仍然艰巨，部分河流治理效果不理想，污水管网不完善，重大环保设施落地难；六是审批环节过多、效率不高、推诿扯皮等问题依然存在，一些工作人员依法行政意识和能力还不强，腐败问题时有发生。我们要进一步强化忧患意识，居安思危、力戒自满，正视矛盾、直面问题，按照市第六次党代会提出的“五破五立”的要求，采取有效措施认真加以解决。

　　二、未来五年的奋斗目标和主要任务

　　各位代表，今年是深圳经济特区建立35周年。在新的发展时期，中央对深圳经济特区寄予厚望。党的十八大后，习近平总书记离京视察第一站就来到深圳，勉励我们改革不停顿、开放不止步。今年伊始，习近平总书记专门就深圳工作作出重要批示，充分肯定了深圳各项事业发展取得的新成绩，要求我们在全面建成小康社会、全面深化改革、全面依法治国、全面从严治党中创造新业绩，努力使经济特区建设不断增创新优势、迈上新台阶。李克强总理年初视察深圳时，寄望特区继续种好国家改革开放的试验田、打造创新发展的高地、成为包容发展的示范城市。这既是对深圳经济特区的巨大鼓舞和鞭策，也赋予我们新的历史使命。我们要牢记使命、勇于担当，解放思想、真抓实干，按照“四个全面”战略布局，在率先上下功夫，在全面上出实招，在整体推进上见成效，勇当“四个全面”排头兵，不辜负中央的重托，不辜负总书记的殷切期望。

　　——勇当全面建成小康社会排头兵，就是要自觉遵循经济规律、自然规律和社会规律，把促进人的全面发展与社会全面进步作为价值取向，把有质量、可持续作为基本要求，始终坚持发展第一要务，牢牢把握战略机遇期，以质量引领发展新常态，以创新增强发展新动能，高标准高质量统筹推进“五位一体”建设，率先实现本市生产总值和居民人均收入“两个翻番”，率先建成较高水平的小康社会，为全国全面建成小康社会作出特区新贡献。

　　——勇当全面深化改革排头兵，就是要把改革作为全面建成小康社会的根本动力，准确把握改革进入攻坚期和深水区的新特征，紧紧围绕社会主义市场经济的改革方向，坚持激发市场潜能、提高政府效能、增强社会自治功能并重，坚持顶层设计与先行先试、整体推进与重点突破相结合，率先在重大改革上引领示范、在难点问题上探索攻坚，形成一批可复制、可推广的制度成果，率先基本实现城市治理体系和治理能力现代化，在深化改革中以特区新作为不断彰显中国特色社会主义制度优越性。

　　——勇当全面依法治国排头兵，就是要把法治作为全面建成小康社会的根本保障，更加注重运用法治方式来维护市场秩序、调节社会关系、规范政府行为、促进公平正义，大力弘扬法治精神、增强法治意识、培植法治文化，率先打造一流法治政府和法治城市，率先形成公开、透明、可预期的法治环境，努力让法治成为城市治理的基本方式，成为全社会的自觉追求，为建设中国特色社会主义法治体系和社会主义法治国家提供特区新样本。

　　——勇当全面从严治党排头兵，就是要牢固树立宗旨意识，深入践行“三严三实”，全面加强政府系统党的建设，严格落实党风廉政建设责任制，持续推进政府职能、作风两个转变，从严治政、廉洁从政，率先建立科学民主的决策机制、落实有力的执行机制、规范严格的监督机制和导向鲜明的激励机制，率先打造为民务实清廉、有限有效有为的现代政府，加快建设廉洁城市，为全面从严治党作出特区新探索。

　　未来五年政府工作的总体思路是：高举中国特色社会主义伟大旗帜，以邓小平理论、“三个代表”重要思想、科学发展观为指导，全面贯彻落实党的十八大以来中央各项决策部署、习近平总书记系列重要讲话和对深圳工作的重要批示精神，按照省委省政府和市第六次党代会部署要求，以“四个全面”战略布局为统领，紧紧围绕“三个定位、两个率先”目标，主动适应引领发展新常态，坚持改革开放创新，坚持深圳质量深圳标准，突出市场化、法治化、国际化、信息化，着力创新发展、融合发展、绿色发展、联动发展、包容发展，推动经济特区建设不断增创新优势、迈上新台阶，努力建成现代化国际化创新型城市。

　　未来五年，我们要全面落实市第六次党代会提出的目标任务，突出重点，创新思路，以强烈的责任担当全力推进更高质量、更可持续的全面发展。

　　（一）深入推进标准、质量、品牌、信誉“四位一体”建设，构建新常态下质量型发展新优势。标准决定质量，高标准才有高质量。要始终坚持深圳质量、深圳标准不动摇，深入践行“三个转变”，把质量作为新常态下发展的第一追求，把标准作为提升质量的基础支撑，瞄准国际一流，构建全面覆盖经济、社会、文化、城市、生态等各领域的质量标准体系，以更高的标准提质量、创品牌、树信誉、拓市场，推动经济持续稳定增长、发展迈向高端水平。深入落实市人大常委会关于加强标准建设若干问题的决定，率先在涉及健康、安全、环保等领域制定实施更严格的标准，前瞻制定一批引领性、创新性标准，探索建立标准试验区，着力打造标准高地，加快从标准的跟随者向引领者转变。实施品牌提升计划，培育一批具有国际影响力的深圳品牌。打造诚信深圳，加强信用体系建设，健全守信激励和失信惩戒机制，让质量标准成为硬约束。到2020年，覆盖国民经济和社会发展各领域的广义质量标准体系基本形成，质量型发展优势更加凸显，本市生产总值达到2.6万亿元左右，人均生产总值3.5万美元左右，努力建成更具辐射力带动力的全国经济中心城市。

　　（二）大力发展信息经济，抢占未来发展制高点。信息经济是继农业经济、工业经济后，以信息技术全面渗透，互联网广泛应用，实现泛在连接和全面智能化的新经济形态。信息经济正在改变人类的时空观，引发生产生活方式和资源配置方式的深刻变革，全面优化重构产业链、创新链、价值链，成为推动转型升级的新动力、促进社会进步的新途径。深圳要主动迎接挑战，捕捉机遇，发挥优势，拥抱信息经济新时代。实施“互联网＋”行动计划，大力推动移动互联、大数据、云计算、物联网等与各行各业相结合，促进虚拟与现实互动、线上与线下整合、技术和产业跨界融合，实现产品个性化、制造智能化、组织多样化、资源云端化，不断创造商业新模式、催生产业新形态。实施“宽带深圳”行动计划，大力推进光纤入户，加快建设“全光网城市”，推动下一代互联网的部署和商用，构建宽带、融合、安全、泛在的新一代信息基础设施，实现感知无处不在、联接无处不在、数据无处不在、计算无处不在，打造“万物互联”的国际一流信息港。实施落实“中国制造2025”行动方案，大力发展机器人、智能装备、传感技术等，加快发展个性制造、柔性制造、智能制造、精密制造，推进智能工厂建设，推动传统制造向高端制造转型，促进信息化与工业化深度融合。

　　（三）积极促进消费、转型、创新“三者互动”，实现产业结构再优化再升级。消费是新常态下实现有质量稳定增长的最大潜力所在。要抓住“两个翻番”带来的消费升级重大机遇，顺应个性化、多样化消费新潮流，坚持需求牵引和供给推动并重，着力从需求方面施策、从供给方面发力，以新需求牵引新技术、催生新产业，以新技术创造新供给、激发新需求，把巨大的消费潜力和创新能力转化为推动产业结构再优化、再升级的强劲动力。着力以消费升级带动产业升级，围绕新需求部署新产业，加强市场和消费心理分析，强化消费政策与产业政策联动协同，瞄准消费结构变化新趋势，组织实施若干消费新热点培育工程，积极培育信息、文化、教育、健康、旅游、养老等领域的新业态，构建与消费升级相适应的现代产业体系，使产业升级的步伐跟上消费升级的变化。着力以供给创新促进需求扩大，破除限制新技术新产品新商业模式发展的不合理准入障碍，支持以技术创新开发新产品、创造新功能，以服务创新降低消费成本、提高消费效率，大力发展智能家居、可穿戴设备、基因诊疗、移动支付等新兴产业，提供更多功能强、品质高、定制式、个性化的产品和服务，挖掘和释放消费潜力。着力打造国际消费中心，围绕高铁、空港、邮轮母港等重要节点，优化商业网点布局，建设品牌展示和体验中心，探索建立全球进口商品交易平台，营造安全舒适的消费环境，形成时尚、创新、品质的消费特色，不断增强对海内外消费者的吸引力，在满足更高水平消费需求中实现升级发展。

　　（四）不断强化创新、创业、创投、创客“四创联动”，努力建成更高水平的国家自主创新示范区。牢固树立抓创新就是抓发展、谋创新就是谋未来的理念，把创新作为引领发展的第一动力，全面落实中央关于加快实施创新驱动发展战略的若干意见，突出开放创新、全面创新和原始创新，统筹推进科技、管理、品牌、组织、商业模式创新，加强知识产权保护，构建更具活力的综合创新生态体系，建设国家全面创新改革试验区。组织实施前沿技术攻关“登峰计划”、创新型企业“倍增计划”和创新型产业空间“扩容计划”，大力发展创客活动，积极打造国际创客中心，全面激发大众创业、万众创新的巨大活力。发挥金融中心城市和多层次资本市场优势，强化资本对创新的支持作用，设立创新创业、新兴产业等大型引导基金，发展创业投资、股权众筹、互联网金融，打造创新型企业融资中心和财富管理中心，推动创新链、产业链和资本链有效对接。构筑创新型人才高地，完善多层次人才政策体系，加大“孔雀计划”实施力度，加快建设一批高水平大学和特色学院，建成国际大学园，探索设立网络大学，推动高等教育开放式、国际化发展。实施高技能人才发展计划，努力建设具有国际先进水平的现代职业教育体系。到2020年，力争国际创新中心建设取得突破性进展，建成10家左右具有国际影响力的重大创新平台，引进高层次创新团队100个，全社会研发投入超过1000亿元，国家级高新技术企业超过10000家。

　　（五）全力落实国家“一带一路”倡议，以湾区经济新发展构建对外开放新格局。要主动谋划、积极作为，充分发挥特区、湾区、自贸区叠加优势，加快打造产业发达、功能强大、开放协同、集聚外溢的一流湾区经济，推动粤港澳大湾区建设和泛珠三角区域合作，努力建成更具竞争力影响力的国际化城市，更好地服务国家战略。加快打造“一带一路”倡议枢纽和海上丝绸之路桥头堡。构建全方位“走出去”支撑服务体系，谋划推进一批重大合作项目，推动与沿线国家和地区交通基础设施互联互通，积极参与信息丝绸之路建设，拓展与喀什等丝绸之路经济带重要节点城市的合作，打通新时期对外开放的战略“双通道”。加快自贸区建设和前海开发开放。落实广东自贸区总体方案，高起点高标准建设前海蛇口片区，加快构建与国际对接的投资贸易规则体系，建成我国金融业对外开放试验示范窗口、世界服务贸易重要基地和国际性枢纽港。深化深港合作，推进前海开发开放大提速，集聚发展高端服务业、新兴服务业和总部经济，促进服务贸易自由化，到2020年前海地区生产总值达到1500亿元左右，成为粤港澳大湾区和海上丝绸之路的重要战略支点。加快提升湾区经济集聚辐射功能。增强金融、科技、人才等高端要素的集聚能力，提升贸易、航运、旅游等产业发展能级，不断扩大湾区经济影响力和辐射力。推动湾区空港、海港、信息港联动，构建人流、物流、信息流、资金流融通汇聚、多元互联的综合枢纽。打造陆海统筹、城海交融的空间格局，形成世界级湾区天际线，彰显一流湾区城市魅力。加快推进粤港澳大湾区合作。全面提升深港、深澳合作水平，主动参与珠三角三大经济圈协调互动，加快深莞惠和河源、汕尾“3＋2”经济圈建设，推进广深港高铁、穗莞深城际线、深中通道、赣深客专、深茂铁路等重大项目规划建设，促进湾区内要素自由流动、产业协同发展、资源高效配置、市场深度融合。

　　（六）狠抓改革攻坚，着力向改革要活力要生产力。深入贯彻落实十八届三中全会精神，进一步解放思想、开动脑筋，突出问题导向、需求导向，自觉把握改革大局、主动服务改革大局，不断推出一批具有全局带动作用的改革举措，积极争取更多国家改革试点，努力建成更具改革开放引领作用的经济特区。加快财税、价格、土地、社区股份公司等改革，深化国资国企、医疗卫生、文化、社会组织等体制机制改革，不断激发市场和社会活力。加大简政放权、放管结合、职能转变力度，进一步深化大部门制、行政审批制度、基层管理体制等改革，理顺市区权责关系，制定实施部门权责清单和市场准入负面清单，强化规划政策引导、事中事后监管、基本公共服务供给等职能，更有效地弥补市场失灵。以信息化技术、互联网思维创新政府服务管理，加快信息互通互认、开放共享，让数据“多走路”、群众“少跑腿”，促进服务和效率“双提升”、职能和作风“双转变”。

　　（七）加快打造一流法治政府，提升城市软实力和长远竞争力。法治是实现治理能力现代化的必由之路。要认真贯彻落实十八届四中全会精神，把法治理念贯穿到城市治理的各个环节，推进法治城市、法治政府、法治社会一体建设，努力使法治成为最显著的城市特质。着力提升依法行政水平，完善法治政府建设指标体系和考评制度，把政府工作全面纳入法治轨道，率先建成法治政府。提高政府立法工作质量，加强政府规章和规范性文件动态清理。健全依法决策机制，推进新型智库建设，建立市政府顾问制度。深化行政执法体制改革，提升基层执法水平。坚持依法治权、以制度管权，推进政务公开，建设廉洁政府。深入开展法治宣传教育，增强全社会法治意识，形成尊法学法守法用法的良好风尚。

　　（八）全面推进绿色化发展，建设更加宜居宜业的现代化国际化城市。深圳是我们共同的家园。要突出以人为本，全面提升城市发展质量，推动发展绿色化、功能现代化、城市国际化。着力加快生态文明建设。全面落实中央关于加快推进生态文明建设的意见，像保护眼睛一样保护生态环境，积极创建国家生态文明建设先行示范区，全力打好治水提质攻坚战，打造绿色港口、绿色交通、绿色社区，使青山常在、清水长流、空气常新。到2020年，PM2.5平均浓度降至30微克/立方米以下，力争实现污水管网全覆盖、主要河流水质达到国家Ⅳ类水标准，公园总数超过1000个。着力推进区域协调发展。实施特区一体化五年攻坚行动，把更多的资源、财力向原特区外倾斜，加快完善基础设施，提升公共服务水平，到2020年基本实现特区一体化。优化城市组团布局，加强土地复合利用。高质量开发建设功能新区和15个重点区域。深入实施城市更新和土地整备，加快城中村、旧工业区和旧商业区改造，加大违法建筑查处力度。着力完善城市服务功能。谋划推进一批重大基础设施，加快轨道交通三期四期、深圳国际会展中心、机场第三跑道和T4航站楼等规划建设。构建国际一流综合交通运输体系，打造全球性物流枢纽城市。推进西江引水工程和海水淡化试点，加强供排水管网建设，切实提高城市供水保障和防洪排涝能力，探索建设雨水自然积存、渗透、净化的海绵城市。着力提升城市国际形象。加强国际交往，发展一批国际友城和友好交流城市，举办更多国际知名展会、高端体育赛事和文化活动，办好第19届国际植物学大会。加快文化“走出去”，推动文化与科技、制造、旅游等渗透融合，提升文博会、文交所、对外文化贸易基地等国际化发展水平，不断打造融入国际元素、体现民族特色的文艺精品，塑造“创意深圳、时尚之城”的国际形象，提升城市文化软实力。

　　（九）坚持发展为民、发展惠民，建成更高质量的民生幸福城市。始终把人民对美好生活的向往作为我们的奋斗目标，满腔热情地做好民生工作，持续加大民生投入，滚动实施重大民生工程、年度民生实事、小康惠民工程，努力增加优质公共产品和服务，让人们生活更有保障，机会更加公平，城市更加包容，社会更加温暖。有序推进基本公共服务均等化。坚持保基本、兜底线、促公平、可持续，更加关心低收入群体和困难群众的生产生活，不断提升社会保障水平，稳步提高最低生活保障标准和最低工资标准。未来五年投入1300亿元，完成养老、安居、文体、食品安全等12项重大民生工程，新增养老床位3000张，建设筹集保障性住房14.9万套。加快提升教育医疗事业发展水平。落实国家中长期教育改革和发展规划纲要，争创国家教育综合改革试验区。提升基础教育办学质量，多渠道增加优质教育资源，五年新增幼儿园学位13.5万个、中小学学位15万个。加快提升民办教育水平，支持特殊教育发展。开展全民素质提升计划，建设学习型城市，进一步提高市民文明素质和人文素养。加快推进智慧医疗、健康深圳建设，大力实施医疗卫生“三名工程”，引进50个以上高层次医学团队，建设80个国家和省级医学重点学科，建成5个名医诊疗中心和10家以上知名医院，打造国际化医疗中心。发展远程诊疗、网络医院、基因检测，扩大家庭医生服务试点，加强公共卫生服务。加强和创新社会治理。继续实施“织网工程”和“风景林工程”，推进基层管理精细化、扁平化，创新社区治理模式。发展公益慈善事业和专业社工服务，建设志愿者之城、爱心之城。规范发展社会组织，构建和谐劳动关系。做好民族宗教和侨务工作。全面实施城市公共安全白皮书，完善城市公共安全防控体系，深入推进平安深圳建设。加强国防教育、国防动员和后备力量建设，推进军民融合深度发展。

　　三、2015年主要工作

　　2015年是落实“四个全面”战略布局的开局之年，也是完成“十二五”规划的收官之年。今年经济社会发展的主要预期目标是：本市生产总值增长8.5%左右，居民人均收入与经济增长保持基本同步，城镇居民登记失业率控制在3%以内，居民消费价格涨幅控制在3%以下。重点做好九个方面工作：

　　（一）着力保持经济有质量的稳定增长。把握稳增长和调结构的平衡点，促进投资、消费、出口更均衡地拉动经济增长。加大重大基础设施、重大民生工程等投资力度，推进15个重点区域开发提速，拓宽民间投资领域，加快轨道交通三期等项目建设，固定资产投资增长14%左右。实施促进商贸流通业发展的政策，推进国家信息惠民和信息消费试点城市建设，培育新的消费增长点。完善外贸稳增长政策，进一步优化外贸结构，加大“一带一路”沿线国家和地区市场开拓力度，扩大先进技术设备、关键零部件等进口，大力发展跨境电子商务、综合外贸服务等新业态，推进国际贸易“单一窗口”试点。落实国家结构性减税政策措施，改善中小微企业融资环境，加大金融对实体经济支持力度。

　　（二）协同推进产业与区域转型升级。研究制定信息经济发展政策，启动实施一批“互联网＋”“宽带深圳”示范工程。大力发展战略性新兴产业和未来产业，产业规模分别达到2.3万亿元和4000亿元。促进现代服务业提质增效，发展创意设计、互联网金融等新兴服务业，引进法人金融机构15家以上。组织实施一批高端制造、精密制造、智能制造示范工程，建成服装、珠宝、钟表、眼镜等14个行业公共服务平台，推动优势传统产业向品牌化、国际化发展。推进高新区北区、保税区、出口加工区等转型升级和华强北改造，完成盐田综合保税区封关验收并正式运行。在家具、服装、电子信息等行业试点开展“深圳标准”认证和标识制度。

　　（三）加快国家自主创新示范区建设。出台贯彻中央关于实施创新驱动发展战略若干意见的具体方案，认真落实全省科技创新大会精神，全面实施国家自主创新示范区规划纲要，激发全社会的创新活力和创造潜能。探索设立未来网络实验室等重大科技基础设施，推进国家能源新材料技术研发中心、先进储能材料国家工程研究中心等建设，新增各类创新载体100家，组织实施重大技术攻关项目60个。引进海外高层次人才团队20个。推进科技服务业区域试点，大力培育研究开发、技术转移、知识产权、专利评估等科技服务业。出台支持创客发展三年行动计划，实施青少年创新专项计划，办好国际创客周。

　　（四）加大重点领域和关键环节改革力度。全面落实三年改革总体方案和年度改革计划，继续争取更多改革试点，把改革新红利加速转化为发展新动能。深化商事制度配套改革和国资国企改革。推动金融改革创新，争取“深港通”尽快获批。实施财政预算管理制度改革，启动中期财政规划，制定第五轮财政体制改革方案。深化土地市场化改革，推进资源类产品价格改革。构建社会组织综合监管体制，加大政府职能转移和购买服务力度。启动医疗服务价格改革，完善医师多点执业配套措施。深化事业单位人事制度改革。加快社区股份公司改革，支持社区经济转型升级。

　　（五）进一步提高开放型经济水平。出台广东自贸区前海蛇口片区具体实施方案，努力形成若干制度创新成果。落实粤港澳服务贸易自由化政策，促进投资贸易便利化。制定落实“一带一路”倡议具体实施方案，充分发挥侨务、商会资源优势和国际仲裁等作用，支持企业参与沿线国家基础设施建设，加强产业合作，争取更多项目纳入国家重大项目库。新增国际友城和友好交流城市5个、国际友好港3个。加快前海开发建设，全年完成固定资产投资160亿元，合同利用外资70亿美元。实施前海促进深港合作工作方案，吸引更多港资港企参与前海开发建设。办好前海合作论坛，深化前海金融创新。加快莲塘/香园围口岸、深圳河第四期治理工程等跨界基础设施建设。深化深莞惠“3＋2”经济圈合作，完成第二轮扶贫开发“双到”任务，积极做好全面对口帮扶河源、汕尾工作，推动援疆、援藏和帮扶毕节、甘孜取得新进展。

　　（六）加强城市建设管理。全面完成新一轮特区一体化三年实施计划，原特区外公交站点500米覆盖率提高至93%，千人总体学位数达到110个。加快轨道交通7、9、11号线建设，力争6、10号线开工，推动8号线建设，推进坂银通道、坪盐通道、外环高速、龙华现代有轨电车试验线、梅观高速市政化改造等项目建设，深化轨道交通四期、大南山隧道、沿一线快速路等规划研究。加强交通综合治理，打通23条断头路，改善南头关等30个片区通行条件，畅通新秀等7个片区交通微循环，优化82个公交站点。完善信息基础设施，加快光纤入户，新建4G基站9700个。推进鹿丹村、白石洲片区等一批城市更新工程。提高市容环境管理和设施建设标准，提升工程建设质量。强化安全生产“一岗双责”，完善突发事件预警信息发布体系，坚决防范重特大安全事故。加强平安深圳建设，健全社会治安立体防控体系，依法严厉打击违法犯罪活动，推动城市公共安全形势稳定向好。

　　（七）持续提升环境质量。落实国家“水十条”，开展治水提质行动，加强茅洲河、龙岗河、坪山河、观澜河、布吉河等河流综合治理，强化对前海湾、深圳湾等污染整治。加大饮用水源保护力度，新开工优质饮用水入户工程80个以上。推进福田污水处理厂、沙井污水处理厂二期建设，新增污水管网300公里以上。加快实施防洪排涝整治计划，完成150个内涝积水点整治。加大新能源汽车推广力度，全面淘汰黄标车。推进绿色港口建设，推广使用港口岸电和低硫油。完成妈湾电厂深度脱硫除尘改造，加强扬尘污染治理，完成重点行业挥发性有机物整治。加快推进垃圾减量分类。积极应用环保新技术、新工艺，提升环境基础设施建设和运营管理水平。新建改造65个公园，开工建设8个森林公园，推进深圳湾滨海休闲带西段建设，实施绿道连通计划。新增绿色建筑面积1000万平方米。加快国际低碳城建设，推进太空科技南方研究院、节能环保产业园等项目建设，办好第三届国际低碳城论坛。

　　（八）加大民生保障力度。今年市财政安排九大类重点民生领域支出1109.8亿元，增长28.7%。完成12项重大民生工程投资200亿元，办好118件年度民生实事，实施民生微实事计划。加大社会保障力度，全面实施重特大疾病补充医疗保险，开工建设保障房1.5万套、竣工1.5万套、供应1.8万套，推进人才公寓建设。新增公办普通高中学位5700个、义务教育学位2.1万个、幼儿园学位2万个。加快香港中文大学（深圳）、南方科技大学、深圳北理莫斯科大学和清华-伯克利深圳学院等规划建设，推进深圳吉大昆士兰大学、深圳墨尔本生命健康工程学院等特色学院落地。与联合国教科文组织合作在深设立高等教育创新中心。坚持产教融合、工学对接，提升职业教育发展质量。大力实施医疗卫生“三名工程”，引进10个以上高水平医学团队，启动名医诊疗中心建设，推动中国医学科学院肿瘤医院深圳医院、南方医科大学深圳医院正式运营，加快新明医院、聚龙医院、市医学科学院等项目建设，新增病床5600张。实施街道医院提质工程，加强基层医疗队伍建设，提升社康中心设备配置标准和服务质量。强化食品和农产品质量安全保障，提高抽检率，依法严厉打击食品药品违法行为。加强公共文化服务体系建设，继续办好深圳读书月、市民文化大讲堂、关爱行动等活动，深入开展全民健身运动。争创新一轮全国全省“双拥模范城”。

　　（九）强化政府自身建设。按照全面从严治党的要求从严治政，加快政府职能和作风转变，简政放权，放管结合，优化服务，建设为民务实清廉政府。坚持依法行政，自觉接受市人大工作监督、法律监督和市政协民主监督，落实重大事项向市人大及其常委会报告和向市政协通报制度，坚决执行市人大及其常委会的决议决定，切实做好建议提案办理工作，认真听取各民主党派、工商联、无党派人士和人民团体的意见，广泛接受群众、媒体和社会监督，加大行政监察和审计监督力度。认真落实中央八项规定，深入推进政府系统党风廉政建设和反腐败斗争。扎实开展“三严三实”专题教育，加强公务员队伍建设，不断增强宗旨意识、忧患意识、责任意识，按照“马上就办、真抓实干、办就办好、滴水穿石”的要求，尽心尽力做好各项工作，努力向全市人民交出一份满意的答卷。

　　各位代表，今年已经过去5个月，我们要全力完成今年和“十二五”目标任务，按照市第六次党代会的部署要求，以改革的精神、创新的理念、科学的方法，凝聚各方智慧，高标准高质量做好“十三五”规划编制工作。

　　各位代表，发展全凭实干，奋斗成就梦想！让我们紧密团结在以习近平同志为总书记的党中央周围，高举中国特色社会主义伟大旗帜，在省委省政府和市委的坚强领导下，解放思想，凝心聚力，奋发有为，不断创造“四个全面”新业绩，努力建成现代化国际化创新型城市，为实现“两个一百年”奋斗目标和中华民族伟大复兴的中国梦，作出深圳经济特区新的更大贡献!
